# Finding the Dieulafoy's Lesion: A Case of Recurrent Rectal Bleeding in an Immunosuppressed Patient

**DOI:** 10.1155/2019/9402968

**Published:** 2019-07-03

**Authors:** Caleb Hudspath, Dylan Russell, Ki Eum, Joel Guess, Jessica Bunin, Franklin Goldwire

**Affiliations:** ^1^Department of Medicine, Tripler Army Medical Center, 1 Jarrett White Road, DHCK-DM, Honolulu, HI 96859, USA; ^2^Department of Surgery, Tripler Army Medical Center, 1 Jarrett White Road, Honolulu, HI 96859, USA; ^3^Department of Critical Care Medicine, Tripler Army Medical Center, 1 Jarrett White Road, Honolulu, HI 96859, USA; ^4^Department of Medicine, Gastroenterology Service, Tripler Army Medical Center, 1 Jarrett White Road, Honolulu, HI 96859, USA

## Abstract

A Dieulafoy's lesion (DL) is rare cause of acute gastrointestinal bleeding defined as a vascular abnormality of the submucosa. With a high prevalence for the stomach and upper gastrointestinal tract, it is rarely observed in the lower gastrointestinal tract. Its prevalence is rare accounting for less than 2% of all cases of acute gastrointestinal bleeding. The etiology of DL is unknown. Common comorbidities include cardiovascular disease, kidney disease, diabetes, alcohol abuse, liver disease, and chronic NSAID use. Few cases involving chronic steroid use and immunosuppressive treatment have been reported. The most common diagnostic and treatment modality is endoscopy with refractory cases treated with arterial embolization by angiography. We present a case involving a patient with significant comorbidities on chronic immunosuppression with a life-threatening, massive lower gastrointestinal bleed from a DL in the rectum.

## 1. Introduction

A Dieulafoy's lesion (DL), defined as a vascular abnormality of the submucosa, is a rare cause of acute gastrointestinal bleeding. Although the mechanism has yet to be fully understood, DL is theorized to result from localized mucosal ischemic damage and acquired vascular ectasia that erode through atrophic mucosa. First described in 1898, DL is mainly found within the proximal stomach; it is rarely observed within the lower gastrointestinal tract. It is associated with a male predominance. A majority of patients have concomitant comorbidities such as coronary artery disease, advanced liver disease, chronic renal disease, and diabetes mellitus [1]. A few cases have been reported in the setting of chronic immunosuppression. Although the prevalence of DL is thought to be underestimated due to the difficulty in diagnosis, cases are being reported more frequently as endoscopy becomes more common [1]. We present a case of rectal DL with findings on both angiography and endoscopy in a middle aged female with known autoimmune disease on chronic immunosuppressive therapy.

## 2. Case Report

A 59-year-old hospitalized female with multiple comorbidities on chronic immunosuppressive therapy (cyclophosphamide and prednisone) for management of Goodpasture syndrome experienced sudden onset large volume hematochezia and hemodynamic instability. Her past medical history is also significant for end stage renal disease on hemodialysis, diabetes mellitus, and anemia of chronic disease. After stabilization with fluid resuscitation and blood transfusion, a technetium labeled red blood cell scan failed to demonstrate evidence of extravascular accumulation and active gastrointestinal bleed. An initial colonoscopy revealed a nonbleeding rectal ulcer with evidence of diverticulosis.

**Figure 1:**
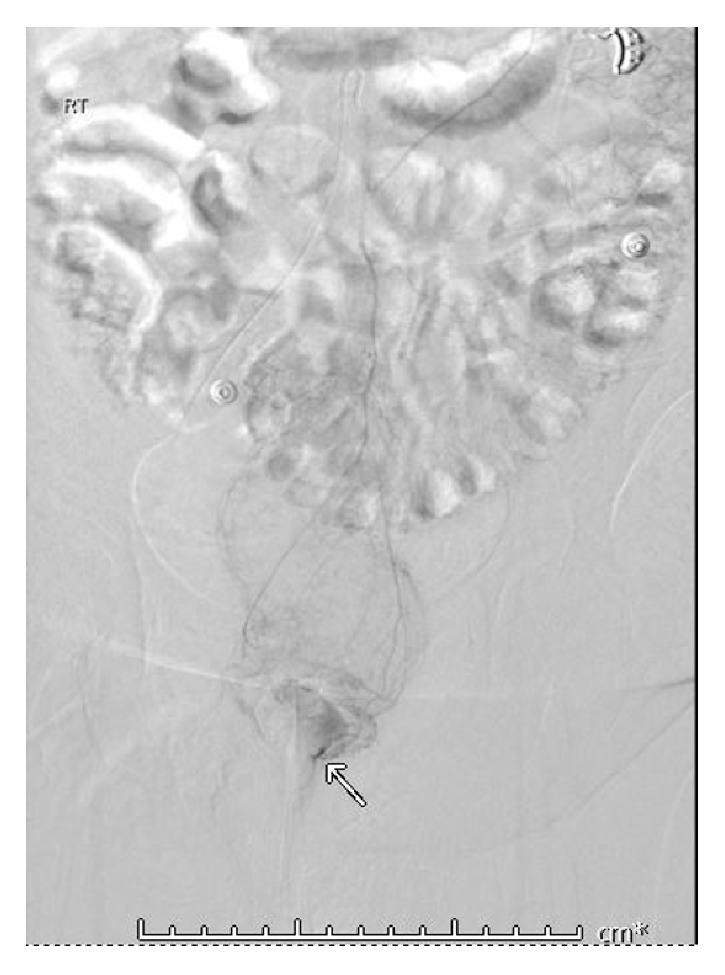
Mesenteric arteriography with contrast extravasation in the rectum.

One week later, the patient presented in hemorrhagic shock due to massive bright red blood per rectum requiring emergent blood product transfusion. Her systolic blood pressure was less than 90 mmHg. Her heart rate was greater than 150 beats per minute. Laboratory findings demonstrated a hemoglobin decline from 8 g/dL to 5 g/dL (normal, 11.7-15.7). Her platelets were reported as 120 × 10^9^/L (normal, 150-440). Physical exam findings were significant for a large amount of bright red blood per rectum with passing of multiple large dark clots. She was given intravenous fluids and thirteen units of blood products with minimal improvement in hemodynamics. Mesenteric arteriography revealed active extravasation in the rectum. She underwent successful Gelfoam embolization (Figure 1).

**Figure 2:**
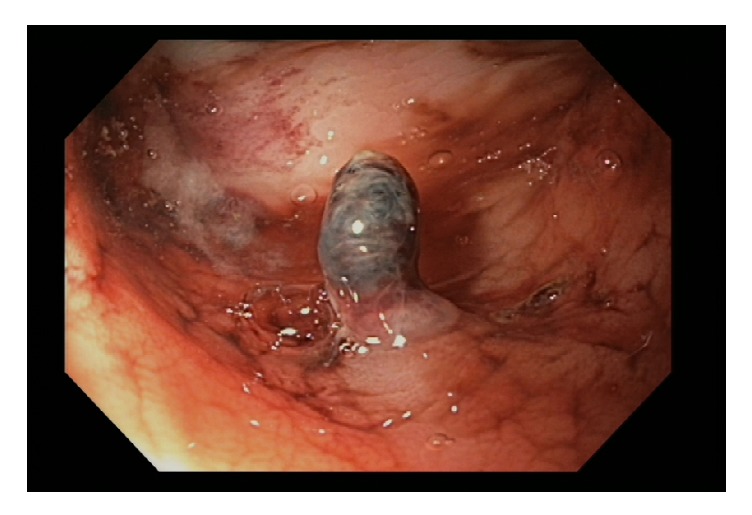
Endoscopic visualization of a single pulsatile artery protruding from the rectal mucosa consistent with a Dieulafoy's lesion.

Follow-up colonoscopy demonstrated a single, pulsatile, visible artery protruding from the rectal mucosa consistent with DL. Mechanical hemostasis was achieved with an epinephrine injection and two hemoclips. A tattoo was placed near the lesion for future identification (Figure 2). The patient experienced no further hematochezia for the remainder of her hospitalization and through a two-month follow-up.

## 3. Discussion

A Dieulafoy's lesion, eponymously named after Paul Georges Dieulafoy who defined the lesion in 1898, is a rare cause of acute gastrointestinal bleeding accounting for 5% of all cases. The lesions are described as arterioles covered by a thin mucosal membrane that become unroofed by some insult (NSAIDs, alcohol, etc.), resulting in rapid blood loss. They are most often diagnosed in the stomach (71%), but are occasionally identified in the esophagus (8%), small bowel (16%), colon (2%), and rectum (2%) [1]. The first recordance of a DL in the rectum was published by Franko et. al. in 1991 and only a handful of cases have been reported since [2, 3].

A DL is an admittedly rare cause of acute lower gastrointestinal bleeding. The majority of lower gastroinstestinal bleeding is caused by diverticulosis (30%-65%) while ischemic colitis, hemorrhoids, polyps, neoplasms, angioectasias, postpolypectomy bleeding, and inflammatory bowel disease constitute the remaining cases. A fraction of all acute lower gastrointestinal bleeding is caused by a DL [4]. According to a study by Schmulewitz and Baillie, the authors reported colonic DL in only 0.09% of all cases of lower gastrointestinal bleeding over a 6-year period [5]. This may be an underestimate as diagnosis can be difficult due to the often small size of the lesion or its tendency to bleed intermittently. The diagnosis of a rectal or colonic DL should be suspected when initial endoscopic evaluation fails to identify a lesion in a patient with recurrent hematochezia and hypovolemic shock.

Both endoscopy and angiography are used in the diagnosis of DL with half being identified on initial endoscopy. An additional third of all DLs are identified on subsequent endoscopy. The current endoscopic criteria used to define a DL include any one of the following: (1) an active arterial bleed or micropulsatile stream from a small mucosal defect, (2) visualization of a vessel with or without active bleeding through a mucosal defect or normal surrounding mucosa, and (3) a fresh, densely adherent clot attaching to a small mucosal defect or normal mucosa [6]. Until 1990, a DL was treated surgically and was associated with an 80% mortality rate. In 1990, Goldenberg et al. suggested that endoscopic treatment should be attempted prior to surgery; this resulted in a substantial decrease in mortality from 80% to 8% [6, 7]. Endoscopic treatment has evolved into a safe and successful modality with a hemostatic success rate reported between 75 and 100% [6]. Angiography is useful when initial endoscopy fails to localize the lesion or the patient is too hemodynamically unstable to tolerate endoscopy. There are no current diagnostic criteria for identifying a DL on angiography but findings of an active bleed include extravasation of contrast into surrounding structures [6].

Our patient's history is notable for active treatment with cyclophosphamide, a potent immunosuppressant. There is at least one other case report of a patient with Henoch-Schonlein Purpura (HSP) treated with cyclophosphamide who died of an unexpected DL bleed. The authors of that case report postulated that a cyclophosphamide-induced pancytopenia increased the hemorrhagic diathesis of their patient [8]. This may have been a contributing factor in this case in which thrombocytopenia was present during the second episode of rectal bleeding. While there is no known direct association between cyclophosphamide use or immunosuppression and lower gastrointestinal bleeds due to a DL, there is a known increased risk of gastrointestinal bleeding in hospitalized patients on corticosteroids (OR 1.42, 95% CI 1.22 to 1.66) [9]. The mechanism by which this occurs is not clearly delineated, but it is thought to be due to impaired tissue repair. Perhaps cyclophosphamide and other forms of immunosuppression similarly impair tissue repair and thus lead to increased rates of DL bleeds. Further research is required to clarify how immunosuppression contributes to risk of a DL bleed.

In conclusion, a DL was diagnosed after mesenteric angiography demonstrated extravasation of contrast into the rectum and repeat endoscopy revealed a nonbleeding pulsatile vessel. This patient's history of chronic renal disease, diabetes mellitus, and Goodpasture syndrome managed by chronic immunosuppressive therapy likely contributed to the development of a DL. This case demonstrates the importance of a maintaining high suspicion for DL in the setting of recurrent gastrointestinal bleeding with multiple risk factors.

## References

[1] Wang M., Bu X., Zhang J. (2017). Dieulafoy's lesion of the rectum: a case report and review of the literature. *Endoscopy International Open*.

[2] Franko E., Chardavoyne R., Wise L. (1991). Massive rectal bleeding from a dieulafoy's type ulcer of the rectum: a review of this unusual disease. *American Journal of Gastroenterology*.

[3] Lee Y., Bair M., Chen H., Lin I., Wu C. (2015). A case of massive lower gastrointestinal bleeding from a rectal Dieulafoy lesion. *Advances in Digestive Medicine*.

[4] Gralnek I. M., Neeman Z., Strate L. L. (2017). Acute lower gastrointestinal bleeding. *The New England Journal of Medicine*.

[5] Schmulewitz N., Baillie J. (2001). Dieulafoy lesions: a review of 6 years of experience at a tertiary referral center. *American Journal of Gastroenterology*.

[6] Jeon H. K., Kim G. H. (2015). Endoscopic management of Dieulafoy’s lesion. *Clinical Endoscopy*.

[7] Goldenberg S. P., DeLuca Jr. V. A., Marignani P. (1990). Endoscopic treatment of Dieulafoy’s lesion of the duodenum. *The American Journal of Gastroenterology*.

[8] Cristescu D. A., Yuvienco C., Schwartz S. (2012). Fatal henoch-schönlein purpura in an adult with dieulafoy lesions. *JCR: Journal of Clinical Rheumatology*.

[9] Narum S., Westergren T., Klemp M. (2014). Corticosteroids and risk of gastrointestinal bleeding: a systematic review and meta-analysis. *BMJ Open*.

